# Principles of synthetic biology

**DOI:** 10.1042/EBC20200059

**Published:** 2021-11-02

**Authors:** Kathryn L. Garner

**Affiliations:** Formerly of Applied Sciences, Northumbria University, Ellison Building, Newcastle-upon-Tyne NE1 8ST, United Kingdom

**Keywords:** metabolic engineering, synthetic cells, regulatory circuits, synthetic genome

## Abstract

In synthetic biology, biological cells and processes are dismantled and reassembled to make novel systems that do useful things. Designs are encoded by deoxyribonucleic acid (DNA); DNA makes biological (bio-)parts; bioparts are combined to make devices; devices are built into biological systems. Computers are used at all stages of the Design–Build–Test–Learn cycle, from mathematical modelling through to the use of robots for the automation of assembly and experimentation. Synthetic biology applies engineering principles of standardisation, modularity, and abstraction, enabling fast prototyping and the ready exchange of designs between synthetic biologists working around the world. Like toy building blocks, compatible modular designs enable bioparts to be combined and optimised easily; biopart specifications are shared in open registries. Synthetic biology is made possible due to major advances in DNA sequencing and synthesis technologies, and through knowledge gleaned in the field of systems biology. Systems biology aims to understand biology across scales, from the molecular and cellular, up to tissues and organisms, and describes cells as complex information-processing systems. By contrast, synthetic biology seeks to design and build its own systems. Applications of synthetic biology are wide-ranging but include impacting healthcare to improve diagnosis and make better treatments for disease; it seeks to improve the environment by finding novel ways to clean up pollution, make industrial processes for chemical synthesis sustainable, and remove the need for damaging farming practices by making better fertilisers. Synthetic biology has the potential to change the way we live and help us to protect the future of our planet.

## Introduction

Synthetic biology seeks to design and build new biology that does useful things. This is not a straightforward task and has only been possible in the last 20 years. It has required a great deal of prior work in understanding how cells and biological systems work, and huge developments in technology – in mathematical modelling, deoxyribonucleic acid (DNA) sequencing, and DNA synthesis. It has also required researchers to work together across disciplines – biologists, engineers, chemists, physicists, computer scientists, and social scientists – to form global networks of people exchanging ideas and working together.

**Figure 1:**
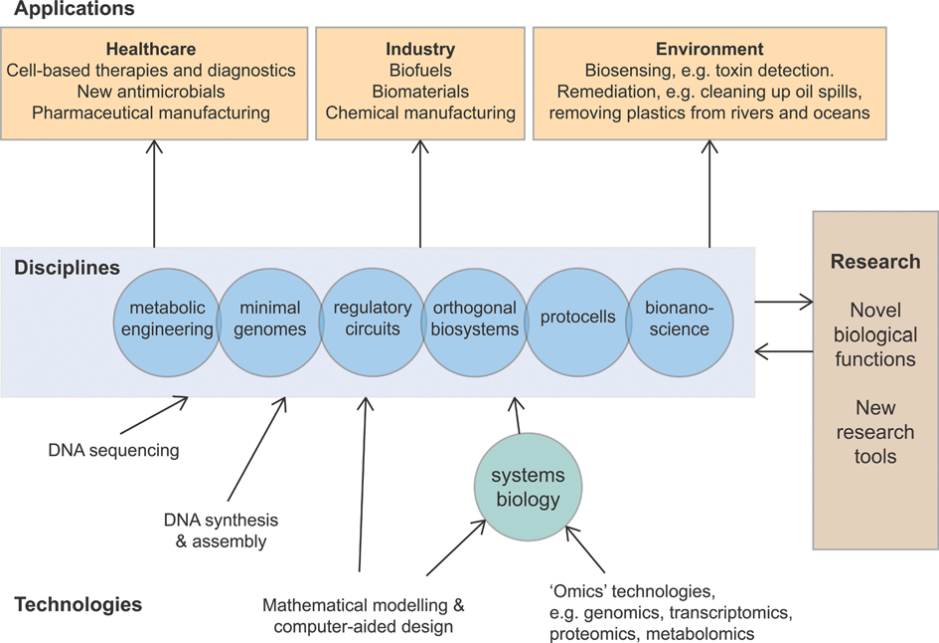
Overview of synthetic biology Technologies underpinning synthetic biology (bottom), disciplines using synthetic biology approaches (middle), and areas of application for synthetic biology products. Ongoing research is key to developing novel biological functions that can be exploited or developed further, and new research tools. A key area of research that contributes to synthetic biology is systems biology.

The sort of useful things that synthetic biology strives to make are far-reaching, and it has been said that by 2030, each one of us will have eaten, worn, used, or been treated with a product made using synthetic biology. Figure 1 provides an overview of the synthetic biology field. Synthetic biology has applications in healthcare, industry, and the environment. Several disciplines use the synthetic biology approach, including metabolic engineering, minimal genomes, regulatory circuits, and orthogonal biosystems, and ongoing research is essential in all disciplines to test new ideas and develop new products and research tools.

For the field to establish itself, sufficient advances were needed in the underpinning technologies. DNA sequencing technology advanced during the massive, international Human Genome Project of the 1990s. Mathematical modelling and the ‘omics’ technologies, such as transcriptomics, proteomics, and metabolomics have progressed along with the systems biology field, which itself has taken a ‘holistic’ approach to understanding how cells and organisms work, meaning that it looks at networks of interactions or systems as a whole rather than focussing on just one or two component parts, as we will see.

The field of synthetic biology has received a huge amount of investment in recent years, and this reflects how significant its contribution to the world is set to be. The industry is reported to have received approximately $7.8 billion in private and public investment in 2020, which is more than twice the funding received in either of the two preceding years, 2019 or 2018. Furthermore, the global synthetic biology market value is estimated to exceed $14 billion by 2026 [1].

In healthcare, synthetic biology can be used to improve diagnosis of disease, and it can be used to make new treatments. An important part of developing new pharmaceuticals is making medicines with fewer side effects. Side effects or ‘off-target’ effects tend to occur if a drug binds to lots of different cells and proteins in the body, despite only a small number of these binding sites being necessary for its disease or infection-fighting property. Synthetic biology can be used to make treatments directed only towards the site of disease, treating the disease itself without causing any negative effects.

Many pharmaceuticals are naturally made by plants. These include pain killers, morphine (an opioid) from poppies and salicylic acid (a plant hormone and precursor to aspirin) from willow bark, but it is not always easy to produce a high yield of these compounds. Synthetic biology can be used to engineer yeast to produce medicines more efficiently, on a large scale, making them also cheap to produce. The first drug to be produced in this way was artemisinin, a treatment for malaria. Artemisinin is produced naturally by sweet wormwood, but the biosynthetic pathway that makes the precursor of artemisinin, artemisinic acid, has been assembled in *Saccharomyces cerevisiae* (*S. cerevisiae*, baker’s yeast) by the pharmaceutical company, Sanofi. The yield of artemisinin had been improved by re-engineering the yeast cells with the genes they need for the pathway. This process, of using genetic engineering to increase or establish a cell’s production of a substance, is known as metabolic engineering.

Kymriah (by Novartis) is the first therapy using engineered living cells to have been approved by the U.S. Food and Drug Administration. It is a treatment for B-cell acute lymphoblastic leukaemia (ALL), a cancer affecting the B lymphocytes (or B cells) of the immune system. B cells are the cells in the blood that make antibodies to help fight infections. ALL is the most common type of cancer in children. Synthetic biology has been used to re-engineer the patient’s own cells to fight their cancer, which is remarkable. The treatment involves isolating the patient’s T lymphocytes (or T cells) – these are the immune cells that detect infection – and genetically modifying these cells to express a chimeric antigen receptor (CAR). CAR is a fusion protein which includes an antibody that recognises CD19 protein, which is present on the malignant B cells. The modified ‘CAR-T’ cells re-introduced into the patient are now able to recognise the cancer cells as malignant. CAR-T cells can then ensure that the cancerous B cells are targeted for destruction by the immune system. CAR-T cells can survive in the patient for years, if not decades, making them a very effective and specific therapy for treating ALL.

It is not just the patient’s body cells that can be engineered to fight disease. We have bacteria that live in our bodies – on our skin, in our mouths, and in our digestive system – and these bacteria are beneficial to our overall health. In our digestive tract, the so-called ‘good bacteria’ work alongside our body processes, enhancing our immune systems and metabolism. Furthermore, these bacteria can also be engineered to fight disease. Patients with a genetic disease called phenylketonuria (PKU) are unable to break down the amino acid phenylalanine. The build-up of phenylalanine causes damage to tissues, particularly the brain. Pharmaceutical company Synlogic has used metabolic engineering to enable the bacterium *Escherichia coli* Nissle 1917 strain to break down phenylalanine into non-toxic molecules, a huge milestone in the treatment of PKU. This treatment is currently in phase 2 clinical trials, which means it has been tested in healthy volunteers with no harmful effects and will now be trialled in sufferers of PKU.

Bacteria that live in soil and water can also be appropriated for synthetic biology. Here, they can be engineered to help to prevent damage to the environment. Throughout the world, farmers add nitrogen fertilisers to their crops to improve yields; plants need nitrogen, and they will grow better if they have the right amount of it. However, nitrogen fertilisers are produced through a chemical manufacturing process that consumes huge amounts of energy. This production process is unsustainable, and the use of these chemical fertilisers is hugely damaging to the environment. Pivot Bio is a biotechnology company that has re-engineered a type of bacteria called γ-Proteobacterium that already associates with cereal crop roots (including corn, wheat, and rice) to fix nitrogen from the air and provide it to the plant roots in nitrate form that the plant can use. Ordinarily, nitrogen-fixing bacteria would not associate with cereal crops. Pivot Bio re-engineered the γ-Proteobacterium to ‘switch on’ nitrogen-fixing pathways that were otherwise dormant to make their product, PROVEN, now available to farmers across most of the United States. In bacteria, switching on genes is achieved by removing repression at a gene promoter; we will be returning to this concept in the Regulatory Circuits section below.

Other products made using synthetic biology enable us as consumers to make our own choice to do our bit for the planet. Many of us acknowledge that eating a vegetarian diet would be better for the environment; rearing animals for food requires large areas of the countryside and keeps biodiversity in the area low. Yet it is difficult to substitute for the taste of meat. Impossible Foods have recognised this and have deduced that it is the iron-containing haem chemical in the blood that is crucial for the experience of eating meat. In response, the company engineered the yeast *Pichia pastoris* to produce soy leghemoglobin, a similar, iron-containing compound, to make their plant-based burgers taste more meaty.

More than half of new clothes produced contain plastics. Plastic fibres include polyester, nylon, acrylic and polyamide, and when clothes containing these materials are washed, they shed millions of plastic microfibres that persist in the environment. Bolt Threads in California have engineered yeast to produce spider silk, which they purify and spin into fibres. In 2019, Stella McCartney revealed their Adidas tennis dress made using a microsilk and cellulose blend fibre from Bolt Threads. Produced in yeast, these fibres are protein-based and therefore are designed to be fully biodegradable.

As we have seen, synthetic biology seeks to design and build new biology that does useful things. It uses living organisms as its medium, but this can make some people feel uncomfortable. The cells and processes engineered can be considered to be ‘unnatural’ and scientists working in the synthetic biology field might be viewed as ‘playing god’. Where should the line be drawn between ‘natural’ and ‘unnatural’? Is it important that we draw a line at all? When synthetic biology products are made that have commercial value, the industry needs to know who owns these products and these ideas, and that is when we talk about Intellectual Property in the legal sense. What does it mean to create and own life? Before we can answer these questions, we should first consider what life is. Later, we will come to understand cells and living processes as machines that can be understood and tinkered with, albeit wet and squishy ones.

## What is life?

The first cells were observed by Robert Hooke using an early microscope, and his ‘Micrographia’, published in 1665, carried the first description of a microorganism. Dutch draper and amateur microscope builder Antonie van Leeuwenhoek was aware of Hooke’s work, and using simple microscopes he had built, examined lots of different natural substances to see what they looked like under a microscope. In 1676, van Leeuwenhoek discovered bacteria, which he referred to as ‘wee animalcules’.

In the 150 years that followed, others confirmed the findings of Hooke and van Leeuwenhoek, but it was some time before microscope technology advanced such that these instruments became widely available. In 1839, Matthias Jakob Schleiden and Theodor Schwann wrote, “All living things are composed of living cells”. It was now understood that the cell is the primary biological structure for life, but where do cells come from?

The popular belief in biology at the time was that of ‘spontaneous generation’. This belief held that life can arise spontaneously from non-living matter – such as fruit flies from bananas, or maggots from rotting meat.

However, some scientists were starting to think otherwise. In 1855, Rudolf Virchow, widely considered to be the father of modern pathology, proposed the ‘Biogenic Law’: *Omnis cellula e cellula*, or “All living cells arise from pre-existing cells”. Together, Schleiden, Schwann and Virchow defined the Cell Theory, which is still widely accepted today. Cell Theory states that:

- all living organisms are made up of one or more cells,
- the cell is the basic biological structure of life,
- cells arise from pre-existing cells.

Indeed, spontaneous generation was sent packing once and for all by Louis Pasteur in 1859 with his famous experiments. Pasteur boiled broth in two glass flasks. One flask was left open to the air, while the other had a neck curved in an ‘S’ shape with a cotton wool plug in the top. As the contents of the open flask cooled, the solution became cloudy as bacteria began to grow within it. The broth in the second flask, however, remained clear – sterile, free of live bacteria. Pasteur had not only disproven spontaneous generation with these experiments, he had also demonstrated that microorganisms were everywhere, including in the air we breathe.

It is important to note here that there were bacteria in the second flask, only that they were not live bacteria. Boiling the broth had killed the bacteria that had ventured into the flask before the top had been sealed. What is the difference between the bacteria in the two flasks, one population alive and the other dead? This leads us to another belief that was rife in the 18th and 19th centuries, one which was much harder to shake, that of Vitalism.

Vitalism was the belief that life required a ‘vital spark’, an ‘energy’ or ‘*élan vital’*. Proponents of vitalism believed that the key to life was a substance distinct from chemical and physical sources. But it might be more appropriate to think of the vital spark in terms of booting up a computer or of Frankenstein giving life to his monster – in terms of the requirement of an organism to absorb energy from its surroundings.

In 1944, Erwin Schrödinger, Austrian physicist and Nobel laureate, published a short book for a general audience, ‘What is Life? The Physical Aspect of the Living Cell’. This book was based on a series of public lectures given by Schrödinger in the previous year and sought to think about life from the point of view of physics.

The second law of thermodynamics tells us that, in the universe, or in any closed system, the degree of disorder can only increase. Leaves raked into a neat pile will be blown by the wind to again cover the lawn. Books, toys, and children’s building blocks tidied away will inevitably cover the bedroom floor again. The universe favours chaos, yet cells themselves are not chaotic. In fact, they are very ordered. Cells appear to defy the second law of thermodynamics because they are ordered.

Metabolic pathways enable cells to capture energy from their environment (see earlier essay in this series on ‘Metabolism’ [2]), and it is the energy from these pathways that cells use to create and maintain their order. A cell itself is not a closed system, and the more energy a cell absorbs from its environment, the more disorder they push out into their surroundings in the form of heat and waste products.

Schrödinger was also the first person to propose that living cells contained a kind of codescript. He suggested that this codescript would have atoms that are well-ordered, and he referred to it as an ‘aperiodic crystal’. He imagined that this crystal would be able to carry enough information within itself to determine the entire pattern of an individual’s future development. It is now clear that the aperiodic crystal that Schrödinger had postulated is DNA. The structure of DNA was announced by James Watson and Francis Crick in April 1953. ‘Aperiodic’ means ‘not regular’, so although the atoms are well-ordered, they vary along the length of the DNA molecule.

We also now understand that DNA is essential to life. Without DNA, cells are unable to make their chemical messenger, messenger RNA (mRNA). They are unable to make protein, they are unable to replicate or evolve. They are unable to replace old, tired, worn-out components and organelles in the cell, and they are unable to make new lipid membranes. If a cell loses its genetic information, it will die in minutes to days. Only red blood cells are able to exist without DNA, lacking both a nucleus and mitochondria, but red blood cells must be frequently replaced by the body.

Having said that DNA is essential for life, of course DNA on its own in a tube is not alive. It needs the environment of the cell with all its metabolic processes surrounding it to exhibit the hallmarks of life – to metabolise, grow and reproduce, differentiate, communicate, move, and evolve. Similarly, a virus is not alive – it needs to enter a cell in order to replicate, and to ultimately itself evolve.

## Biological systems and systems biology

In the previous section, we discovered that cells use metabolic pathways to capture energy from their environment, and that they use this energy to create and maintain order. These metabolic pathways are themselves ordered, typically a series of enzyme reactions that at each step take the product of a prior reaction in the pathway and convert it into the next component in the pathway. Pathways rarely operate in isolation, and it is more usual to think of a pathway as part of a larger network or system of pathways, with shared components and multiple crossovers.

Cells also use pathways of interconnecting components to perform ‘housekeeping’ roles, the processes necessary to keep life running smoothly. These include recycling old cell components or organelles and making new components (the latter is called ‘biosynthesis’ or ‘biosynthetic pathways’, and it is particularly useful to make use of these pathways for producing chemicals and other compounds in synthetic biology).

**Figure 2:**
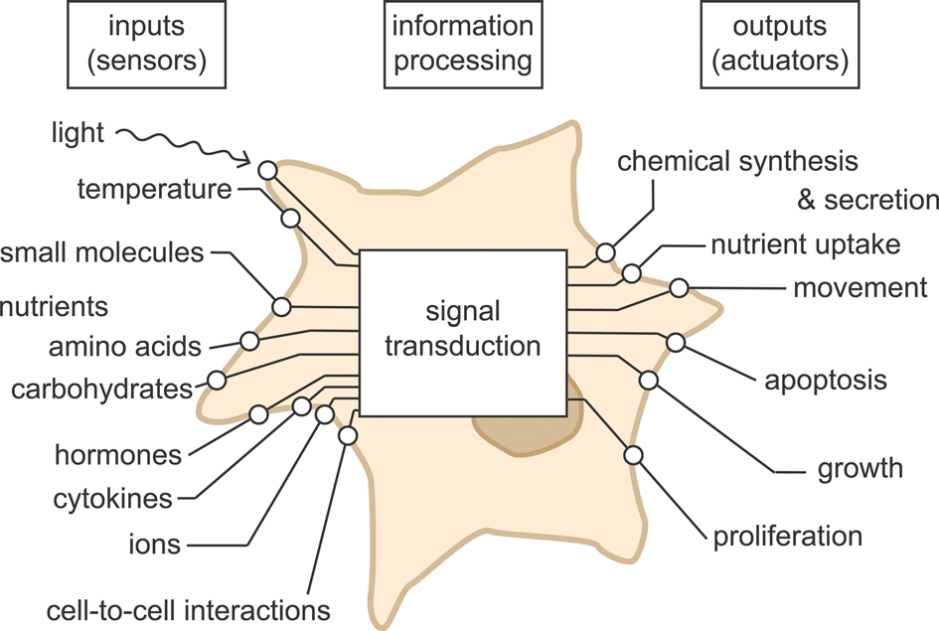
Cells sense and make sense of their environment Cells continually sample their environment. Depending on the cell type and array of receptors (sensors) displayed on their surface, cells monitor their environment for nutrients, light, cell–cell interactions, and communication signals in the form of hormones and cytokines. This information is communicated and processed through signalling pathways, enabling the cell to respond appropriately (output through actuators).

Another important feature of a cell is its ability to sense and make sense of its environment. Cells use appropriate receptors (sensors) on their surface to survey their environment (Figure 2). Depending on the cell type and array of receptors they present on their surface, they might look for small molecules, such as hormones and nutrients. In response to the hormone insulin, muscle cells in animals take up glucose from the blood to store as glycogen, and they do this by sending glucose transporters to the cell surface to permit the uptake. A single-celled alga, such as *Chlamydomonas reinhardtii*, uses sunlight to provide its energy through photosynthesis, and so detection of light by its sensors directs the organism to move towards the light source. Other signals arrive through cell–cell interactions. Sensors such as these can be made use of in synthetic biology for making systems that respond to their environment, discussed particularly in the ‘Disease Sensing’ section below.

Information a cell receives about its environment and internal state must be processed to enable the cell to respond appropriately. It does this using signalling pathways and networks, and the complexity of these processes mean that cells can be thought of as complex information-processing systems, comparable with computers and other electronic devices.

**Figure 3:**
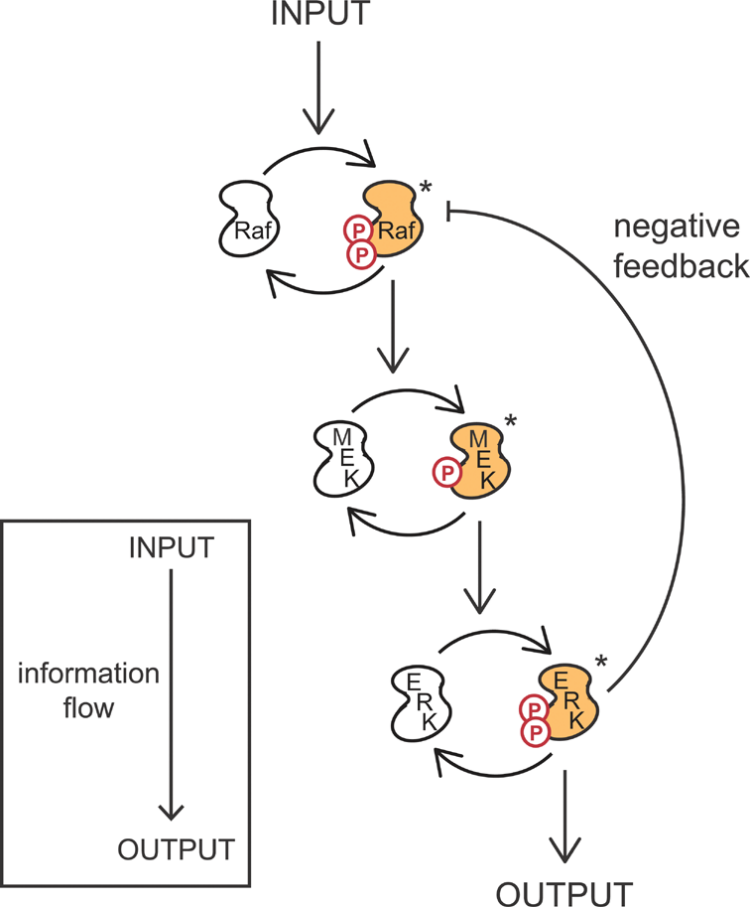
The MAP kinase signalling cascade MAP kinase pathways consist of three kinases, shown here are Raf kinase (MAPKKK, MAPKK kinase; phosphorylates MAPKK), MEK (MAPK/ERK kinase, MAPKK; the kinase of MAPK), and ERK (extracellular signal-regulated kinase, MAPK, MAP kinase). Phosphorylation activates the next kinase in the cascade. Information is transduced from Raf to ERK, and negative feedback regulation occurs through phosphorylation of Raf by ERK in minutes to switch off signalling. In addition to phosphorylating Raf, active ERK translocates to the cell nucleus to activate gene transcription. * indicates the active enzyme.

Signalling pathways are composed of a series of protein–protein (or sometimes protein–lipid) interactions. Each interaction moves the signal on from one component to the next, and promotes a chemical change in the second component, activating it and so permitting it to contact and modify the next component in the pathway. The mitogen-activated protein (MAP) kinase cascade is the most common signalling pathway in eukaryotic cells (Figure 3). It consists of three enzymes called kinases that each attach a phosphate molecule to the next kinase in the pathway in a process called phosphorylation. This is the most common form of ‘post-translational modification’ used to regulate signalling pathway components. At each step, the addition of the phosphate is the activating signal for the next kinase in the pathway, and at the same time, phosphatases are working to remove the phosphates to switch off the signal, keeping the signalling processes tightly controlled.

Regulation of signalling can also be achieved through simple feedback loops, such as the one shown for the Raf-MEK-ERK pathway in Figure 3. Once extracellular signal-regulated kinase (ERK) is activated through phosphorylation by MAPK/ERK kinase (MEK), ERK translocates (moves) to the nucleus to activate gene transcription, but it also phosphorylates Raf at a different site to the activating phosphorylation to deactivate it. This process is known as negative feedback and occurs in a timescale of minutes, limiting the duration of the signal that can pass through the pathway. This is a means to fine-tune a pathway. We do not completely understand all of the signal processing that occur inside cells, but it is understood that a changing input signal (for example, short pulses) carries more information than a single maximal signal, and that cells know how to decode these changing inputs.

Negative feedback is also important in biosynthetic pathways, where the product of the pathway feeds back to switch off its own production, a way of saying that enough of the molecule has been made and production can stop.

Positive feedback, by contrast, serves to reinforce and amplify a signal. Here, the output of the signalling pathway feeds back to further activate upstream pathway components, which can ensure that the signal is robust to minor variations or fluctuations in the input signal. A positive feedback loop with an ultrasensitive regulatory step can produce a bi-stable switch. Under basal conditions (this could be resting conditions or unstimulated – in the absence of a signal from the environment), the system is said to occupy its basal state. The system, however, is constantly sampling its environment – it is ultrasensitive to any change in signal. For inputs below a threshold value, the system remains in the basal state. However, when the input signal climbs over a certain threshold value, the system switches to the second state. Both states are stable, and it is the signal from the environment that causes the shift between states. This type of system also holds memory, as the current state stands as a record of the signal that took it to that state.

Simple feedback loops can combine in different ways to produce different biological outcomes, and these are called emergent properties. Combinations of negative and positive feedback loops can create oscillations, where the output of the system changes over time. These oscillations can be pulses or waves, and might only occur in a distinct region of a cell, as is common in calcium signalling. Setting up local regions of signalling molecules can cause polarisation of a cell, for example when it is required to move in a particular direction. This occurs when a neutrophil (a phagocytic white blood cell like a macrophage) is attracted towards a site of inflammation, with signalling molecules accumulating along the edge of the cell leading the movement.

**Figure 4:**
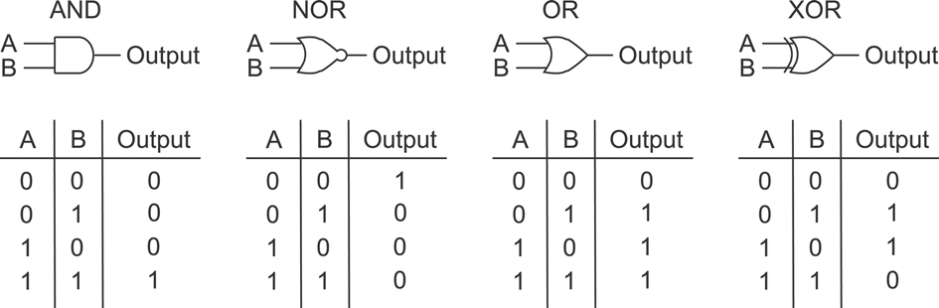
Signal integration by logic gates receiving multiple inputs Various types of digital two-input gates. The standard symbol used to represent each type of logic gate is shown at the top, and how the output of the gate depends on the state of each of the inputs (A and B) is shown in truth tables at the bottom. Inputs and outputs are either ‘off’ or ‘on’, denoted by ‘0’ or ‘1’.

To understand how cells process information, it can be useful to make comparisons with human-made digital control systems. Such systems depend on logic gates, which specify an output, or response, depending on the combination of two inputs. Each input or output is assumed to be ‘off’ or ‘on’ and given the symbols ‘0’ or ‘1’. A variety of logic gates with outputs are shown for two inputs in Figure 4, and their names correspond to Boolean operators, AND, NOR, OR, and XOR. In a simple two-input AND gate, both inputs are required to produce the output. An example of an AND gate is signal processing by Akt (Protein kinase B, PKB), which can be phosphorylated at residues Thr^308^ and Ser^473^. Akt is fully activated by phosphorylation at both sites to enable it to activate downstream signalling components and therefore is analogous to an AND gate.

Systems biology as a field seeks to understand these complex information-processing systems, and has driven advances in high-throughput technologies that enable huge datasets to be generated comprising hundreds or even thousands of measured outputs. These huge datasets can themselves be difficult to understand in their entirety; to help with this, systems biologists have worked alongside mathematicians, statisticians, and computer scientists, and have become adept at applying information theory and developing mathematical models. Systems biology seeks to understand the way that cells are wired as information-processing systems; synthetic biology uses this knowledge to design and build its own systems.

## Mathematical modelling and computer-aided design

Signalling networks are complex and the datasets generated are so vast that it is necessary to use computers to make sense of the data. In metabolic engineering, some chemicals are easier to produce in yeast than in bacteria, yet a single yeast cell has 2000 metabolic reactions, which are associated with more than 900 genes. Mathematical modelling can aid in understanding how these reactions connect to one another, by processing the data and abstracting it to make the networks of reactions easier to visualise.

Mathematical models can be constructed using either deterministic or stochastic approaches. In a deterministic model, the system is described through a series of mathematical equations, usually a set of Ordinary Differential Equations (ODEs). These equations include an indication of the rate at which each reaction will happen (the rate constants are parameters), and the relationship between system components, with relative component concentrations defined as variables. This type of modelling can be useful, for example, to understand how negative feedback can shape signalling through the MAP kinase pathway.

At the molecular level, signalling pathways are composed of hundreds or thousands of individual components, each one of which is subject to random molecular diffusion. This randomness is referred to as ‘stochasticity’; mathematical models can be constructed that account for this randomness, referred to as stochastic models. Modelling individual molecules can be particularly revealing when the numbers of molecules involved is very small; in this situation, small differences in molecule behaviour can lead to significant changes in the system output.

By abstracting data and modelling system components, we can learn more about features of a system that would not have been apparent through observation alone. Many biological systems are oscillatory, they act like a pendulum or a wave rising and falling over time. Oscillatory systems include the concentration of hormones like insulin and the tandem glucose oscillations in the blood, the circadian clock, and calcium dynamics within cells. Until mathematical modelling was used, however, we did not know that non-linearity (the change in the rate of reaction or information flow in a system), feedback, and a time delay are necessary to produce an oscillator. In this way, mathematical modelling can be used to test hypotheses, make predictions, and carry out experiments *in silico* (meaning in a computer simulation) relatively quickly, limited only by available computing power.

In addition to mathematical modelling, computers can be used to optimise the design of experiments. A suite of methods is available to tackle this, known as Design of Experiments (DoE). Computers can be used to automate building and testing of synthetic systems using robots across large scales in multi-well experiment plates. Using robots and computers in this way enables lots of different options for biological designs to be tested very quickly (rapid prototyping) and optimised. Automation removes the contribution of human error and bias in experiments, and makes experiments more reproducible.

## Engineering life

Earlier, we introduced words familiar in engineering, talking about cells as information-processing systems with sensors and actuators. One of the main features of synthetic biology is that it applies engineering principles to biology. It utilises engineering’s concepts of standardisation, modularity, and abstraction to promote rational design for industrial application. DNA is the code for building a part (a biological part or ‘biopart’), parts are used to build biological devices, and devices can be combined and assembled into biological systems.

Standardisation occurs across the field, from standard working methods and standard ways to represent biological designs, to defining standard bioparts. Synthetic biology promotes an open way of working, where researchers are encouraged to collaborate and share models, designs, and resources. This simplifies the design and build process, and makes it faster, as scientists can use and enhance what others have done, rather than having to start from scratch with each build. This process also enables further rigorous testing and reduces bias in experiments – having another scientist take your designs and use them in their own system provides further reassurance that what you saw is reproducible and applicable for the field to use more widely.

**Figure 5:**
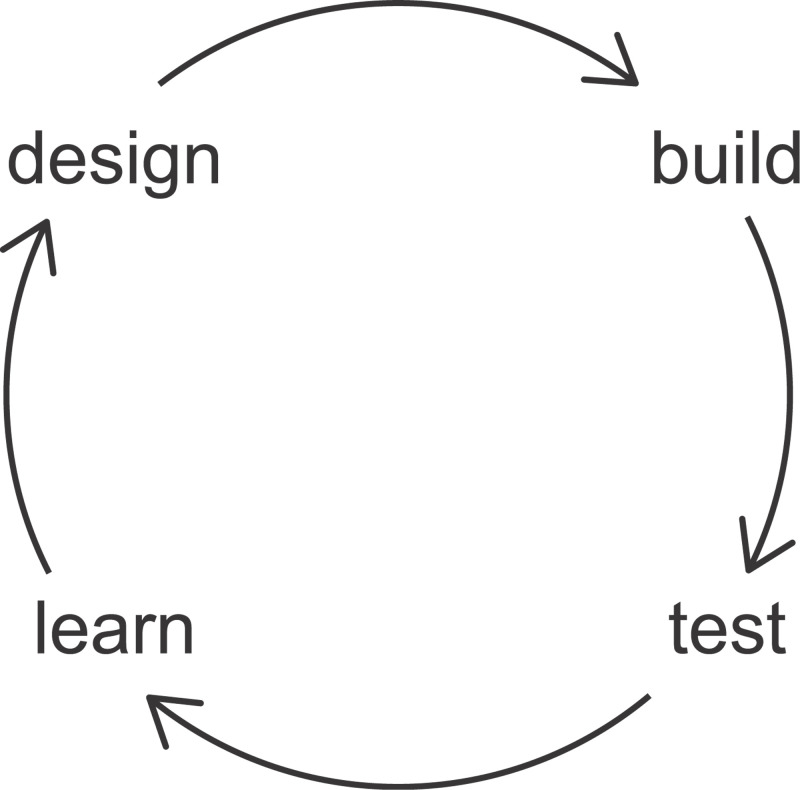
Working process in synthetic biology Making new biology typically follows iterative cycles of Design, Build, Test, and Learn.

Working methods follow a characteristic cycle (Figure 5), again drawing parallels with the engineering cycle used in making new machinery or electronics. The first stage in creating a new biological system is the Design stage. This may include making a detailed computer model and considerable testing of the system *in silico*. Early simulations of how the system will perform can be compared with how the real system performs after it has been built (in the Build stage). The differences between what is predicted and what is observed in the Test stage can provide new information (the Learn stage). This new information can be used to modify and refine the model in a new Design stage. This cycle can go through multiple iterations before the desired new biology is attained.

Researchers in the field like to quote Nobel prize-winning theoretical physicist, Richard Feynman, who once said, “What I cannot create, I do not understand”. Each turn of the Design–Build–Test–Learn cycle brings the scientists one step closer to realising their new synthetic system, yet it also enables them to learn more about the underlying natural biology. What better way is there to learn how something is constructed than to try to build it yourself?

**Figure 6:**
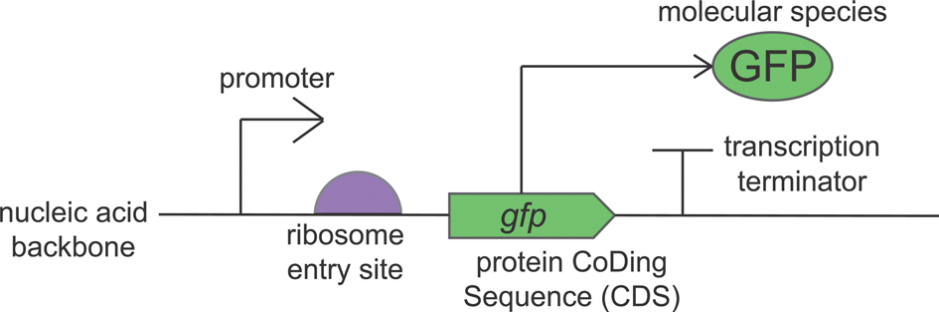
SBOL Visual Standard description of a bacterial protein-coding gene made using SBOL Visual.

To enable designs and data to be shared amongst the synthetic biology community, a data standard has been developed, called Synthetic Biology Open Language (SBOL). This data standard provides a method of representing biological designs *in silico*. Its goal is to make data exchange between scientists more efficient. SBOL data models can be written in a variety of true programming languages, such as C++, Java, or Python. It also provides a standard for describing biological systems visually, called SBOL Visual. The standard glyphs (or symbols) for transcription of a bacterial gene, described using SBOL Visual, are shown in Figure 6. As in circuit diagrams used in electronic engineering, here elements of the genetic machinery have been simplified and abstracted.

To ensure that bioparts can be used to build biological devices, and for these devices to be combined and assembled into systems, it is imperative that bioparts can easily fit together as modules, with only minimal amounts of optimisation. If you buy a second set of toy building blocks to expand your collection, you want to be sure the second set conforms to the same specifications as the first set so that they fit together.

Bioparts, devices, and biological systems often need the environment of a cell to make them work. In particular, the cell is needed to provide energy to the biopart and to mediate inputs from outside the cell. In synthetic biology, the environment of the cell can be provided by an ‘off the shelf’ standard part – referred to as the chassis. The chassis provides the energy to run the synthetic system and is often thought of as the ‘hardware’ of synthetic biology, and the bioparts and biological devices thought of as the ‘software’. The bioparts are ‘loaded and run’ in the chassis. Through modularisation of parts, the goal is ‘plug and play’ biology.

A registry of standard biological parts is held by the BioBricks Foundation and makes biopart designs available to the public free of charge. In this registry, bioparts are referred to as BioBricks™. This Foundation is a not-for-profit organisation founded by engineers and scientists from Massachusetts Institute of Technology (MIT), Harvard, and University of California San Francisco (UCSF) alongside the iGEM competition (see later ‘Creative Education’ section). Each BioBrick is described on a datasheet (or specification sheet) comprising a set of parameters and performance characteristics. PhytoBricks is a similar registry of standard bioparts set up for plants.

Examples of bioparts are regulatory circuits, which are designed to perform a logical function and include sensor, counter, and timer elements. Two specific examples are described in the dedicated section below. Circuits such as these are designed to be ‘tuneable’ meaning that, once combined into devices and systems, small adjustments can be made (like turning the dial on a radio) so that the systems respond to a particular, desirable range of inputs; for a particular range of inputs, the system gives an appropriate range of outputs. This might mean that the outputs match with the technology available to monitor them – the amount of ribonucleic acid (RNA) produced by the system is not so small that it can barely be detected; the amount of fluorescence produced is not so high that it bleaches all of the images, where the signal exceeds the range for accurate and reliable detection.

## Chassis

*Escherichia coli* (*E. coli*) has been used as the model microbe in molecular biology research for the last 60 years or so, and for this reason it is the go-to chassis for synthetic biology. It is referred to as a ‘laboratory workhorse’; it is easy to maintain (the medium on which it grows is cheap and easy to source), it grows in conditions that are easy to replicate in the laboratory (a temperature of 37°C), and it grows fast, with a doubling time of 20 min. The *E. coli* genome (the entire content of its DNA) has been completely sequenced; it harbours a single chromosome approximately 4.6 Mb (megabase pair (1 Mb = 1 000 000 bp)) in length, comprising approximately 4000 different genes. Synthetic gene circuits can easily be introduced into the bacterium’s cytoplasm using DNA plasmids through the process of transformation. An alternative bacterium used as a chassis is *Bacillus subtilis* (*B. subtilis*). Thirdly, yeast, such as *S. cerevisiae* used in winemaking, brewing and baking, is a eukaryote, and therefore when used as a chassis it is more appropriate for thinking about our human cells. *E. coli*, *B. subtilis*, and *S. cerevisiae* all grow in liquid suspension, meaning they can be grown at scale in large bioreactors.

The host chassis will determine whether or not the bioparts work as they are intended. It needs to be well-characterised and understood, with limited unwanted interactions to enable the novel system to work as cleanly and efficiently as possible. It might be that a particular biopart would work better in one chassis than in another. Although the genomes of *E. coli*, *B. subtilis*, and *S. cerevisiae* have all been sequenced, there is still much we do not understand about the way these organisms work. Ongoing research offers a ‘top-down approach’ to uncovering their minute biological function, and what we learn depends on the context and the questions asked. To circumvent this problem, some scientists have suggested that the answer is to design a chassis from scratch, from the ‘bottom-up’. Creating a chassis in this way would enable all of the processes within it to be modelled, defined, and controlled completely.

## Minimal cells

An ideal chassis would be one with the smallest number of genes necessary to support metabolism, and this is termed the ‘minimal cell’. Efforts to define a minimal cell have been made largely by scientists working at the J. Craig Venter Institute (JCVI) in the United States. They began by looking at *Mycoplasma genitalium* (*M. genitalium*), the bacterium with the smallest known genome of any free-living organism, which causes infection in humans by living in the urinary tract downstream of the kidney.

The team first created a synthetic copy of *M. genitalium*, 582 970 base pair (bp) in length, which they named *M. genitalium* JCVI-1.0. DNA synthesis technologies have advanced such that the ability to order synthetic DNA from gene synthesis companies is commonplace today. DNA lengths in the region of 3000–5000 bp are routinely made as standard, and some companies offer DNA synthesis up to 12 000 bp in length. In fact, the limiting factor for the length of DNA that can be synthesised is the length of DNA that can be accommodated by your chosen vector (see earlier essay in this series on recombinant DNA technology for more on DNA vectors [3]). *M. genitalium* JCVI-1.0 was assembled from synthetic fragments, 5000–7000 bp in length. These small fragments were combined into progressively larger fragments, 24, 72 kb (an eighth of the genome), and 144 kb (a quarter of the genome), within a bacterial artificial chromosome (BAC) in *E. coli*. From here, the four quarters were transferred into *S. cerevisiae*, whose expression vectors are able to accept much larger DNA inserts. The team later demonstrated that a synthetic *Mycoplasma* genome was able to support life.

In order to minimalise the genome, the JCVI scientists compared the genomes of bacteria from different genera – *E. coli*, *B. subtilis, M. capricolum*, and *Haemophilus influenzae* – to deduce which genes could be expendable. They identified that bacteria have several genes that can perform the same or interchangeable functions, which is referred to as redundancy. Carefully defining the media used to grow the synthetic bacteria enabled further genes to be dispensed with; where the bacteria could survive on either glucose or fructose sugars as their food source, genes for both glucose and fructose membrane transporters would be present. However, by having only glucose in the cell culture media, only the glucose transporter gene was required, and therefore the gene for fructose transport could be removed. In 2016, the team announced their Minimal Cell, with a chromosome 531 kb (kilobase pair) in length comprising just 473 genes.

## Cell-free

Although a chassis provides the hardware necessary to run the synthetic system, it can be difficult to control and can lead to unanticipated side reactions. For some purposes, it may be possible to remove the cell environment altogether. Methods have been developed that combine just the cell components necessary to drive the system in a test tube. We refer to reactions carried out in a test tube in the laboratory as ‘*in vitro’*, from Latin meaning ‘in glass’. The cell components may be crude extracts, or they may be purified proteins, perhaps themselves made using bacteria or in a cell-free system. An example of a reaction performed *in vitro* with purified components is the polymerase chain reaction (PCR), used to amplify a DNA sample sufficiently to enable it to be studied (see earlier essay in this series [3]).

Biological cells are bounded by a lipid cell membrane or cell wall. Within the cell membrane, the cytoplasm is further subdivided by lipid membranes to create organelles. These compartments each have their own function and identity, but they also serve to increase the effective concentration of enzymes by reducing the area over which they can diffuse. Compartments also group together subsets of cell components, making it more likely that they find each other, and react or pass a signal from one component to the next.

Removing the cell membrane enables direct access to the inner workings of the cell. A substrate required for a reaction no longer needs to be transported across the cell membrane but instead can easily be added to the test tube. Similarly, products of reactions can easily be removed and purified. The state of the system can be monitored and rapidly sampled. It is possible to generate membrane-bound compartments in a cell-free system by immobilising or tethering the enzymes to a support within the test tube or even to a microchip. Enzymes can be immobilised in geometric formations to enable spatial control of reactions.

In addition to the removal of side reactions with the chassis, a cell-free system is useful because it can easily be scaled up to create a viable industrial process, essential in chemical synthesis operations, for example in making pharmaceuticals.

**Protocells** lie somewhere between a cell and a cell-free system. They are simpler than a natural cell and contain only the components essential to run the synthetic biopart. Like a cell, their contents are bound by a lipid membrane. The goal of protocells, however, is to create primitive cells capable of self-replication. Protocells really are synthetic life forms, unlike natural biology.

## Regulatory circuits

**Figure 7:**
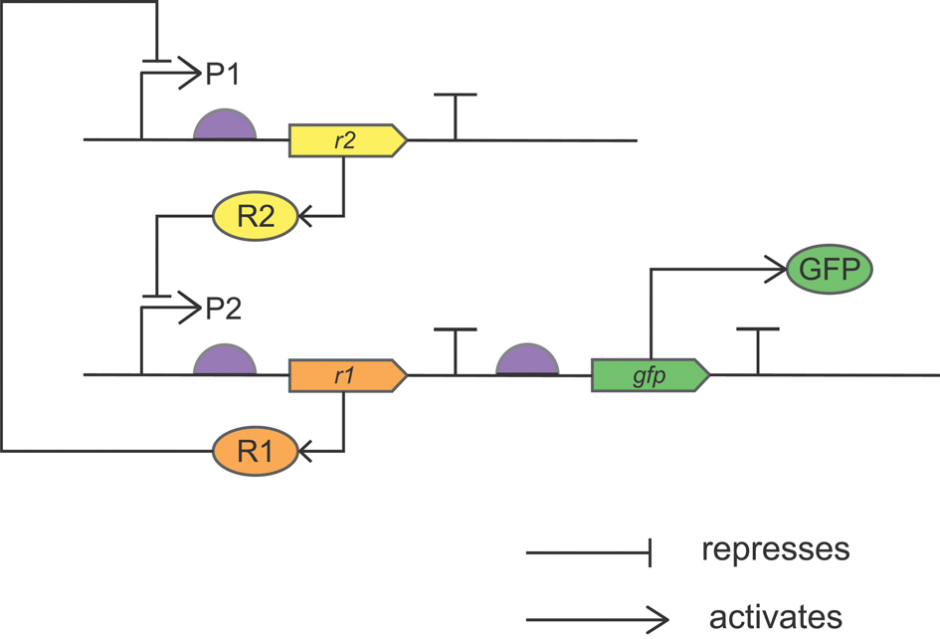
The genetic toggle switch The genetic toggle switch designed and built by Gardner, Cantor and Collins, drawn using SBOL Visual. This synthetic circuit combines two repressor motifs, as discussed in the main text. P1/P2, promoters; *r1*/*r2*, genes encoding repressor proteins; R1/R2, repressor proteins; P2 drives expression of *r1* and also *gfp*, the latter of which is translated into GFP, the system read-out. Both repressor motifs reside on opposing strands of the same double-stranded DNA plasmid, but they have been drawn here separately for clarity.

Synthetic regulatory circuits are bioparts designed to perform a logical function, comparable with the way that electronic circuits work. We saw how logic gates are used in signal processing by cells in the ‘Biological Systems and Systems Biology’ section above. Gardner, Cantor and Collins designed and built a genetic toggle switch from two repressor motifs (Figure 7). Each repressor motif has its own promoter, which is a DNA sequence upstream of the gene to which the enzyme RNA polymerase binds to initiate gene transcription. In this synthetic circuit, a repressor (R1) produced by the first repressor motif (*r1*) blocks transcription from a promoter (P1), which would otherwise drive expression of a second repressor motif (*r2*). Likewise, repressor R2 blocks production of R1 by binding to P2. Expression of green fluorescent protein (GFP) downstream of *r1* enabled the state of the system to be ascertained at any one time – if *r1* was active and *r2* repressed, GFP was synthesised and the cells fluoresced green; if *r*2 was active and *r1* repressed, the cells would not fluoresce. As predicted from the modelling, the system exhibited bistability: either one promoter or the other was active, but not both, and the cells were unable to switch between the two states randomly. Instead, the circuit required an external input to disrupt it and to enable a switch to the alternative stable state, such as the addition of a chemical in the environment of the cell or a change in temperature beyond a threshold value.

**Figure 8:**
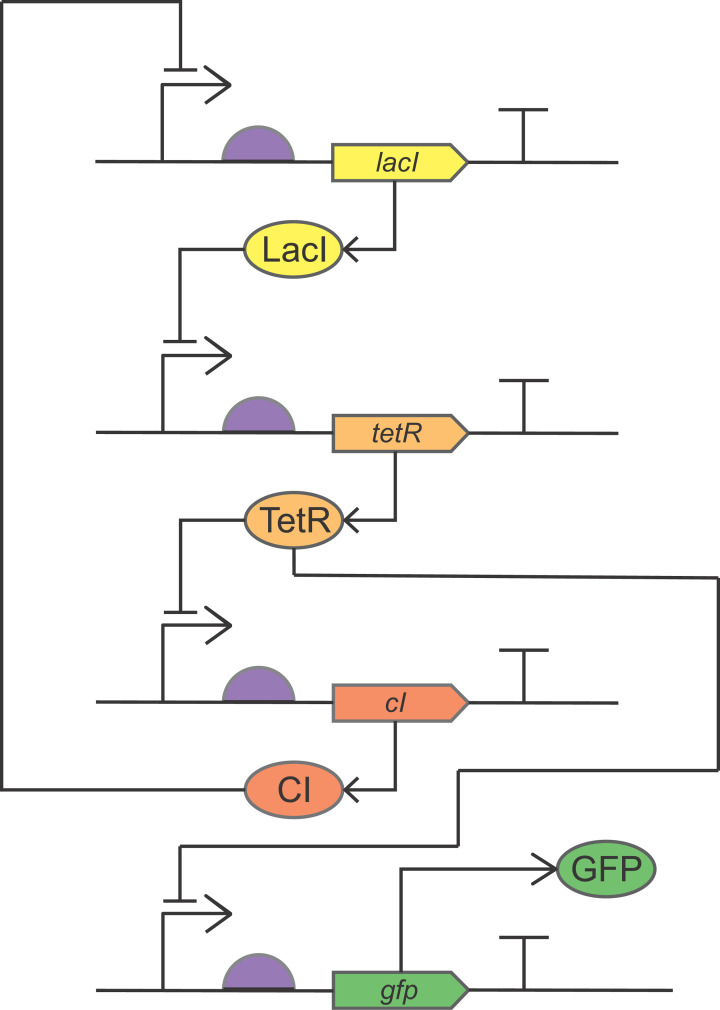
The repressilator The repressilator (ring oscillator) designed and built by Elowitz and Leibler, drawn using SBOL Visual. Comprises three repressor motifs, each taken from a different type of DNA sequence or organism: LacI from *E. coli*, *tetR* from the tetracycline-resistance transposon Tn*10*, and *cI* from λ phage. *lacI*, *tetR*, *cI*, and *gfp* are protein-coding genes; LacI, TetR, CI, and GFP are the protein products. The three repressor motifs are organised in a ring on a repressilator plasmid, and *gfp* is located on a separate reporter plasmid, but they have been drawn here separately for clarity.

The second study, by Elowitz and Leibler, combined three repressor motifs to produce a ring oscillator or ‘repressilator’ (Figure 8). In this synthetic circuit, the product of the first motif blocks expression through the second motif; the product of the second motif blocks expression through the third; and the product of the third motif blocks expression through the first. As with the genetic toggle switch, the state of the system could be read from the presence (or absence) of GFP expression. However, rather than one of the promoters driving expression of GFP downstream of its repressor motif, this time one of the repressor proteins, when expressed, additionally repressed GFP expression. GFP was therefore expressed, and the cells fluoresced green continuously (constitutively), except when a particular one of the repressors was made.

As predicted from the modelling, the repressilator oscillated. An oscillating system can be thought of as a wave that rises and falls according to the amount of GFP expressed. In this case, the amount of time the system took to move from the top of one wave to the top of the next (the period of the oscillation) was several hours. As we noted above, *E. coli* grows and multiplies very fast – a colony will double in size in 20 min, which is faster than the repressilator’s period of oscillation – meaning that the state of the system was passed to the daughter cells at each cell division.

## Disease sensing

We have seen how regulatory circuits can be constructed to perform logical functions, but how might these be used to fight disease?

Circuits can be built able to detect all sort of biomolecules (nucleic acid, carbohydrate, lipid, protein) through appropriate sensors. They can be tuned to detect, for example, a marker of a urinary tract infection, perhaps a protein expressed on the surface of the disease-causing organism (the pathogen); when this is detected, information passes through the synthetic circuit to an actuator, driving expression of a fluorescent protein such as GFP. Simple circuits like this can easily be adapted to detect other pathogens.

Sensory circuits can be run in the traditional sense, as a biopart in a chassis such as *E. coli*, but considerable progress has been made in generating these circuits as cell-free systems. Furthermore, it is possible to freeze-dry sensory circuits on to paper or other porous materials to make systems that are stable for long-term storage at room temperature. This brings huge benefits for disease diagnosis where resources might be sparse, for example, out in the community or in developing countries. These circuits are easily re-activated by soaking with water. The absence of the cell membrane for the cell-free system makes it easy to apply a sample, such as blood or urine, for testing.

Paper-based sensors have successfully been used to detect glucose, which needs monitoring regularly in diabetic patients. They have also been adapted to detect 24 different markers of the Ebola virus. Not only could the Ebola sensors distinguish between the Sudan and Zaire strains of Ebola, the sensory circuits gave results in less that 12 h for just over $20 per sensor, which is favourable compared with existing antibody-based methods for diagnosis. The output of these sensory circuits is often a fluorescent reporter, but some sensors have been designed that produce enzymes that generate colour changes visible to the naked eye, useful in an environment in which a fluorescent microscope might be difficult to come by. The nature of the paper-based sensors also make it possible to combine the sensors with custom-built, low-cost electronics to quantify and automate the detection and diagnosis of disease.

## Synthetic genomics

Protein crystallography has enabled DNA transcription and mRNA translation to be described in terms of the shapes of the molecules involved. Scientists have used this information to design new molecules to fit into these shapes, without chemically reacting with or altering the natural biological system. The new, synthetic molecules use the same systems and principles, and perform comparable activities, without changing the natural biology – this new biology takes place alongside. This concept is called **orthogonality**, and the new coding systems being designed are called orthogonal coding systems.

DNA contains four bases or nucleotides, adenine (A), cytosine (C), guanine (G), and thymine (T), and the sequence or pattern of these bases defines a gene – one gene is the code for one protein. This genetic code is first ‘read’ by RNA polymerase and transcribed into mRNA. The mRNA is translated to protein at the ribosomes – the mRNA bases are recognised in triplets (one ‘triplet’ of DNA bases is called a codon) by a transfer RNA molecule (tRNA). There are 20 essential amino acids, each has its own corresponding tRNA. Each codon codes for a single amino acid. As the mRNA is read, each tRNA brings its amino acid to the growing polypeptide chain in sequence (see earlier essay in this series on nucleic acids [4]).

As there are four nucleotides, this triplet code provides 4^3^ combinations or possible codes for 64 amino acids. However, since there are only 20 amino acids, there is much redundancy. This means that several codons signify the same amino acid, and this means that there is room within the genetic code for engineering new amino acids and increasing the storage capacity of DNA.

In 2013, a team from Yale and Harvard demonstrated that a bacterial genome could be ‘recoded’ by using site-specific mutation. The *E. coli* genome had been made naturally, only the researchers made 321 point mutations in the sequence to remove all occurrences of the codon that is used least by *E. coli* for protein synthesis. Since there is redundancy in the genetic code, these point mutations did not change the amino acid that was being coded for, only the DNA triplet that was used to instruct the tRNA. This organism was named GRO, Genomically Recoded Organism. GRO differed from almost all of life in not using the same 64 codons to direct which amino acids are used to make its proteins. GRO only used 63. It was anticipated that the ‘spare’ codon could be used to add a synthetic amino acid into proteins. This showed it was possible to change the DNA that encodes the protein without altering the protein itself.

The number of codons used by an organism was further reduced in a later study led by Jason Chin at the Medical Research Council Laboratory of Molecular Biology, Cambridge, U.K. (MRC-LMB). In 2019, it was reported that another variant of *E. coli* had been made. This time, simple corrections replaced every known occurrence of two sense codons and a stop codon. In total, 18 214 codons were re-coded to create an organism with a genetic code that used just 61 codons – 59 of which encoded the 20 essential amino acids. Furthermore, this group of researchers were able to remove a whole tRNA, one that was previously thought of as essential. This paves the way for being able to design and implement a purely synthetic tRNA. Such a synthetic system would also require a novel RNA polymerase to insert the non-natural bases into mRNA. A new tRNA would mean that a new amino acid could be designed and used by the cells, but also would require a synthetic aminoacyl-tRNA synthetase to attach the appropriate amino acid to its corresponding tRNA.

In February 2019, a team in the United States announced that they had created an organism that had four synthetic bases in its genetic code, increasing the number of DNA letters to eight. This organism also used the four bases present in nature, but with these additional bases, the ‘DNA alphabet’ had been doubled. They named their synthetic DNA, ‘Hachimoji DNA’. ‘Hachimoji’ means ‘eight letters’ in Japanese. This work was partly funded by National Aeronautics and Space Administration (NASA) as it provides insight into what alternate systems for life might exist on other planets, again demonstrating the power in synthetic biology research for developing potential new industrial applications alongside understanding how fundamental biological systems work (or might work).

In addition to Hachimoji DNA, the researchers designed and made Hachimoji RNA, to enable the DNA instructions to be read by the cell. It was important that the new nucleobases were the same size and shape as naturally occurring adenosine, thymine, cytosine, and guanine nucleotides so that they would be accepted by enzymes already present to replicate the DNA. The group searched for T7 RNA polymerase variants able to transcribe the complete set of Hachimoji nucleotides, rather than needing to engineer their own RNA polymerase. The new bases were able to support life, including potentially all aspects of Darwinian selection, evolution and adaptation. By doubling the genetic code, Hachimoji DNA had increased the density of information that can be stored in DNA.

These studies learning to re-write the genetic code have been conducted in prokaryotes, in bacteria. Bacteria has a single chromosome, so what are the additional challenges faced when we turn our attention to eukaryotes?

The genome of *S. cerevisiae* is 12 Mb in length, organised across 16 chromosomes. The goal of the international synthetic yeast genome project (Sc2.0) is to build a synthetic eukaryotic genome. A highly modified design was specified in 2017, and a consortia of research groups around the world are involved in building the synthetic chromosomes. The design involved some recoding, as well as some minimalisation. Non-coding elements, such as transposons and introns, were removed, and the genome has been ‘defragmented’, with all tRNA genes removed from their usual locations on the main chromosomes and moved to their own new dedicated tRNA chromosome. Use of the word ‘defragmenting’ references the process by which files on a computer hard drive are moved and re-ordered so that all related files are located together, improving data storage. TAG stop codons have been recoded to TAA to allow insertion of non-natural amino acids.

Surprisingly, it has been found that the yeast genome is able to accommodate large structural rearrangements without too much difficulty. Two teams have shown that the 16 yeast chromosomes can be fused together in two very long chromosomes without being detrimental to the yeast cells. Fusing these two chromosomes together into a single chromosome was also able to support a living yeast cell, albeit one that grows more slowly. By contrast, such large rearrangements have not been tolerated so easily by bacterial cells.

In addition to the deletion of non-essential genomic elements and the large-scale reorganisation of genes, special regions of DNA, called *loxPsym* sites, have been inserted into the 3′ untranslated region of all non-essential genes. These sites enable a process referred to as SCRaMbLE, to take place after regions of each chromosome have been synthesised. The addition of an enzyme, Cre recombinase, activated by β-estradiol, causes deletions, insertions, and inversions to be made in the genome at these sites. This permits the rapid shuffling and optimisation of the yeast genome, ultimately allowing its rapid evolution.

The Sc2.0 project can be used to answer a wide range of questions about genome architecture, the fundamental properties of chromosomes, gene content, and the function of RNA splicing and small RNAs. The SCRaMbLE system allows novel robust yeast strains to be generated and screened for practical uses, such as industrial fermentation of agricultural products into biofuels or producing enzymes that could have important applications in medicine.

The Human Genome Project-Write (HGP-Write) is an open, international project aimed at synthesising human genomes. As with other synthetic biology projects, the goal of HGP-Write will be to understand the human genome by building a synthetic copy of it. The synthetic genome will be executed in cell lines (not multicellular organisms), with one aim being to make a human cell line resistant to all viruses.

## Creative education

International Genetically Engineered Machine (iGEM) began in January 2003 as an independent study course at MIT, in which students developed biological devices to ‘make cells blink’. In the following year, iGEM became a Summer competition that has since grown to include over 350 teams in 2019 from more than 40 countries. The competition is open to high school, undergraduate, and graduate teams. In the annual Giant Jamboree, teams compete for the coveted BioBrick trophy.

Teams begin by choosing their own synthetic biology project, either building on previous projects, or defining something new entirely. At the start of the Summer, the teams are given a ‘Distribution kit’ of bioparts (BioBricks) including promoters, terminators, reporter elements, and plasmid backbones. They have 10 weeks to use these parts, together with new parts that they have designed, to build novel biological systems in living cells.

The iGEM competition can provide a test-bed for new ideas in synthetic biology. Student participants gain experience in scientific research, creative design, science communication, teamwork and networking. iGEM is closely supported by industry, and many teams go on to start companies later on. Ginkgo Bioworks was founded by advisors to the 2006 MIT iGEM team. The team’s *Eau d’e coli* project made *E. coli* strains that smelled like banana and wintergreen, and this led to Ginkgo’s early success with the development of yeast strains to produce fragrances, flavours, and food for commercial use.

## Responsible research

Synthetic biology aims to create new life forms, which is a huge responsibility and could have ethical, safety, and security implications. Tools for doing synthetic biology are becoming more accessible, and the growth of the internet has enabled information and ideas to be freely exchanged. The field and what is possible is constantly shifting, and it is important that potential issues are constantly talked about to tackle any problems that arise. Several international working groups have been set up to ensure guidance and regulations remain current and applicable.

Responsible Research and Innovation (RRI) is a term used by the European Union to ensure that people carrying out scientific research and developing new technologies are thinking about the potential impact that their work will have on the environment and on society. As we have seen above, synthetic biology has the potential to benefit the whole of society, yet society will also suffer if things go wrong. It is therefore important that as many different kinds of groups of people and viewpoints are brought into discussions about synthetic biology, and it is important that they are included throughout projects, from applications to gather funding, through to realisation of the final project. Involvement from social scientists, ethicists, artists, and patients are key for ongoing discussions.

Ash Toye at the University of Bristol studies red blood cells. His group is genetically manipulating and culturing red blood cells, and clinical trials infusing healthy volunteers with cultured cells derived from adult blood are currently underway. A blood transfusion might be needed during an operation or following an injury, or a patient might need a transfusion if their own body is unable to make enough blood. The Bristol trial is focussing on producing enough blood for patients with conditions which affect the haemoglobin (the iron-carrying chemical) in the blood. Diseases such as thalassaemia and sickle cell anaemia are typically found in relatively small ethnic populations, and therefore there can be a shortage in providing enough blood to treat these patients. Expanding blood stocks using cell culture techniques could meet this need, but how would patients and the public feel about receiving blood that had been grown in a lab? To explore this, Ash Toye worked with artist Katy Connor and social scientists, Maria Fannin and Julie Kent, to explore patient and public attitudes (see ‘Recommended Reading’). It is important those who stand to benefit most, patients and the public, are listened to, to ensure these opportunities are exploited to their fullest.

At the end of the ‘Introduction’, we approached the term Intellectual Property. Synthetic biology aims to create new biology that does useful things, and because these things have been designed and made by people, Intellectual Property rights are assigned to them. Some people feel that the ‘unnaturalness’ of synthetic biology products distinguish them from natural biology, and therefore feel that it is acceptable or appropriate that they should be owned and bought or sold. Others feel that the concept of ownership is at odds with the open way of working that the field of synthetic biology promotes. Some fear that large corporations will buy up all of the ownership rights to synthetic organisms, creating a monopoly on how they can be used.

Assigning ownership undoubtedly has the advantage of assigning responsibility. A common theme in science fiction is for a synthetic organism made in a lab to escape containment and to wreak havoc on the world. If someone created and therefore owns that organism, they are ultimately responsible for keeping it contained, and they can be held accountable if it escapes. Usefully, when generating their minimal cell, the JCVI team added a stretch of DNA that contained a code that they referred to as a ‘watermark’, or barcode, enabling them to label and track their synthetic organism if needed. A watermark such as this can also be thought of as an ‘I made this’ stamp, like a signature on a painting or piece of pottery.

It can be argued that cell cultures are often difficult enough to maintain in a lab, and it is therefore highly unlikely that a synthetic organism would be able to survive outside of such a controlled environment. Engineering synthetic cells that are only able to survive on a defined media containing a small selection of key nutrients serves as a ‘kill switch’ for laboratory cells or ‘fail-fast’ mechanism, ensuring that the organism is unable to survive without that media. However, synthetic cells designed to clean up plastic or oil pollution in rivers and seas, or synthetic cells designed to circulate and hunt out disease in the human body might not so easily be controlled.

Synthetic cells inside the human body can be engineered with a kill switch that responds to a change in temperature. These cells would be stable and proliferating at 37°C, but as soon as the temperature drops, as would occur if they were excreted from the body, the cells could be programmed to commit suicide. Other environmental factors can be used as monitors, sensed in this way, such as the amount of salt – a synthetic organism might survive in the natural salinity of the sea, but be killed if it ventures into fresh water. Alternatively, it may need to be in contact with hydrocarbons if tasked with cleaning up an oil spill. The synthetic organism could be programmed to die once the spill has been dealt with and it is no longer in contact with the oil. There is a fear that synthetic organisms that have been sent out into the environment could pick up mutations that change their function, causing infection to humans and animals and damage to the environment. If this is foreseen, another kill-switch might be engineered in the organism that produces a toxin to kill the organism if mutations in its genome are detected.

So far, we have discussed ways to curb the accidental release of synthetic organisms. With the rise in do-it-yourself (DIY) culture surrounding synthetic biology, in which biological experiments are executed outside of normal research environments, communications with schools and clubs and other organisations are required to make sure that the understanding of relevant regulations is spread adequately to reduce the risk of malicious use.

Improved DNA sequencing technologies have meant that huge quantities of gene sequence data are now freely available in online databases. Long stretches of DNA can be synthesised quickly and cheaply, meaning that gene and genome synthesis is becoming available to all. Biosecurity concerns have now been triggered by the synthesis of several pathogenic (disease-causing) viruses. In 2005, the virus that caused the 1918 influenza pandemic was reconstructed from sequenced material recovered from frozen lung tissue samples. Prior to this, in 2002, Cello, Paul and Wimmer at State University of New York, Stony Brook, announced that they had assembled a synthetic poliovirus genome from the published sequence alone. Poliovirus is an RNA virus, with a genome ∼7500 nucleotides in length, but the researchers first made the complementary DNA sequence by fitting together smaller overlapping fragments, then transcribed this back into viral RNA. Through this work, the scientists demonstrated that it is no longer necessary to have access to natural pathogens to be able to make a biological weapon (bioweapon). Many gene synthesis companies now perform self-governance, screening DNA orders for known dangerous sequences.

## Current challenges

In reality, the uptake of standards amongst the synthetic biology community is low due to the difficulties in standardising complex biological systems. Quite often, we just do not know enough about individual gene function to use them successfully in engineered circuits. Modules frequently do not work the same way in a different system, and further parameters are needed to be altered and optimised to enable the module to work efficiently. Sometimes two synthetic modules in a cell will compete for the same resources, which can confuse and make the outputs seemingly chaotic.

There are clear limits to the amount of synthetic DNA that can be loaded and run in a given chassis. Cells have a limited pool of resources required for biological processes, such as replication, transcription, translation, and metabolic reactions. The addition of a synthetic biopart will undoubtedly put an unnatural extra load on the cell’s activities, leading to slower growth rates and lower protein synthesis rates. This unnatural extra load is referred to as ‘burden’. Starvation stress caused by limited nutrient resources can increase the rate of spontaneous mutations in an *E. coli* genome, ultimately causing genome instability.

Limiting stress and burden needs to be accounted for in the design process. This can be achieved partly through incorporating additional negative feedback loops. The expression of components in the synthetic pathway could be reduced if levels of stress in the cell become too high. It is also possible to split biosynthetic pathways between different bacterial strains in order to reduce their metabolic burden on the cell. Some processes are very costly in terms of protein components and energy expenditure, and the value of high-cost processes should be weighed against that of low-cost processes. For example, powering a yeast cell by fermentation is of low cost, whereas running a yeast cell on respiration brings with it a high cost, since oxidative phosphorylation pathway components are costly to produce and to maintain.

Technological challenges remain for the size of plasmid, and therefore size of DNA insert, or genomic insertion that can be accommodated by different cells. The transfer of whole chromosomes between cells presents a great challenge. This problem is particularly difficult for eukaryotic cells where DNA is linear and specifically modified by methylation. For synthetic genome transfer, these large DNA molecules may have to be packaged using molecules such as histones (the chief protein components of chromatin) that act as spools around which the DNA winds. The other major challenge in genome transplantation is to understand the role of DNA methylation and histone modifications. These modifications directly affect gene activity through gene silencing, and such modifications may play key roles in activating transplanted genomes.

## Summary

- Synthetic biology seeks to design and build new biology that does useful things.
- The field applies engineering principles of modularity, standardisation, and abstraction to promote rational design for industrial application.
- DNA encodes bioparts, bioparts are combined to make biological devices, and devices are built into biological systems.
- Synthetic biology is working towards a ‘plug-and-play’ concept, in which off-the-shelf bioparts will be able to be loaded and run in finely characterised chasses.
- Synthetic biology has applications in healthcare, industrial processes, and for improving the environment.
- Creating new life comes with it a huge responsibility. Ethical, safety, and security implications need to be considered and continually re-assessed.

## Abbreviations

ALL: acute lymphoblastic leukaemia
bp: base pair
CAR: chimeric antigen receptor
DNA: deoxyribonucleic acid
ERK: extracellular signal-regulated kinase
GFP: green fluorescent protein
iGEM: International Genetically Engineered Machine
JCVI: J. Craig Venter Institute
kb: kilobase pair (1 kb = 1000 bp)
MAP: mitogen-activated protein
Mb: megabase pair (1 Mb = 1 000 000 bp)
MIT: Massachusetts Institute of Technology
mRNA: messenger RNA
NASA: National Aeronautics and Space Administration
PCR: polymerase chain reaction
PKU: phenylketonuria
RNA: ribonucleic acid
SBOL: Synthetic Biology Open Language
